# Avoidance of capture by motion onset

**DOI:** 10.3758/s13414-026-03232-9

**Published:** 2026-02-10

**Authors:** Jahnavi Nair, Xiaojin Ma, Richard A. Abrams

**Affiliations:** 1 Department of Psychological & Brain Sciences, Washington University in St. Louis, St. Louis, MO USA; 2 Department of Psychological Sciences, University of Missouri, Columbia, MO USA

**Keywords:** Attentional capture, Inhibition, Visual search, Visual attention, Motion onset

## Abstract

Recent research has shown that salient items can sometimes be suppressed or ignored during visual search. The findings may help resolve apparently contradictory findings in the attention literature. Suppression of salient items, however, has been shown almost exclusively for color singletons, despite the fact that many other visual attributes can also render an item to be salient. We show here, for the first time, that attentional capture by motion onset can also be prevented. In Experiment 1, using an additional singleton task in which suppression is not expected, we confirmed that irrelevant motion onsets are salient and can impair visual search performance. In Experiment 2, we included motion-onset distractors in the search for a prespecified shape. In that task the motion-onset distractor had no effect on performance, indicating that attentional capture by it had been avoided. Finally, in Experiment 3, we replicated the results from Experiment 2 and additionally included probe trials that also revealed avoidance of motion-onset capture. The findings help to generalize and provide support for theories of attentional suppression and avoidance of capture by salient stimuli.

Every day humans must filter out the less relevant things in their visual world in order to more effectively process the more relevant ones. Unfortunately, a salient item, such as one with a unique color or one that is flashing or moving, whether relevant or not, can often capture our attention (e.g., Abrams & Christ, 2003; Christ & Abrams, 2006; Jonides & Yantis, 1988; Theeuwes, 1991, 1992, 2004, 2005). On the other hand, salient stimuli can sometimes be ignored or actively inhibited, especially if they are irrelevant to the observers’ goals (Becker et al., 2010; Folk et al., 1992, 2002; Lien et al., 2008). Whether “stimulus-driven” or “goal-driven” is a more apt characterization of visual attention has been the focus of considerable research. Recently, some researchers have suggested that the apparent conflict might be resolved by the existence of a mechanism that permits top-down goals to override bottom-up salience under certain circumstances (Chang & Egeth, 2019, 2021; Gaspelin et al., 2015, 2017; Gaspelin & Luck, 2018a, 2018b, 2018c; Hamblin-Frohman et al., 2022; Ma & Abrams, 2023a, 2023b, 2023c; Sawaki & Luck, 2010). Those and other researchers have reported that visual searches are often not impaired, and may even be facilitated by the presence of an irrelevant salient color singleton in an array of items to be searched (Gaspelin et al., 2015; Ma & Abrams, 2023b). A theory proposed to account for this is the *signal suppression hypothesis*, according to which salient items do have a high tendency to capture attention—but such capture may be actively suppressed with inhibitory control driven by top-down goals^1^ (for a review, see Gaspelin & Luck, 2018c, Gaspelin et al., 2025). Whether avoidance of capture by salient elements is mediated by an attentional mechanism that actively downweights salient distractors is the focus of ongoing work and debate (e.g., Chang & Egeth, 2019, 2021; Hamblin-Frohman et al., 2022; Oxner et al., 2023)—but regardless of the conclusion, it is clear that there are certain conditions that permit people to avoid the adverse effects of salient distractors. Learning more about the conditions that permit reduced processing of salient distractors is an important part of understanding attentional mechanisms more generally, and that is our goal here.

In the last decade, suppression of salient distractors has been thoroughly studied, with converging evidence coming from a variety of techniques: psychophysical studies (Gaspelin et al., 2015; Ma & Abrams, 2023a, 2023b, 2023c; Stilwell & Gaspelin, 2021; Vatterott et al., 2018), eye tracking (Gaspelin et al., 2017, 2019; Ipata et al., 2006; Zhang & Gaspelin, 2024), and electrophysiological measurements (Gaspar & McDonald, 2014; Gaspelin & Luck, 2018a; Hickey et al., 2009; Jannati et al., 2013; Ma et al., 2025; Sawaki & Luck, 2010; Stilwell et al., 2022; for a review, see Gaspelin et al., 2023). The vast majority of these studies have manipulated saliency by creating a discontinuity in the color dimension. In particular, many studies have used a color singleton (the sole item of one color among an array of homogenously colored items of a different color) as the salient item (e.g., Gaspelin et al., 2015). As a result, our understanding of distractor suppression is restricted to salience in the color dimension, while many other types of salience have also been shown to capture attention. In particular, items that have a unique size (Becker, 2010; Kiss & Eimer, 2011) or are moving (Hillstrom & Yantis, 1994; McLeod et al., 1991; Yantis & Egeth, 1999) may also be highly salient. A recent attempt to broaden the scope of the type of salience in distractor suppression was made by Adams and Gaspelin (2024), who compared the ignoring of static feature singleton distractors with dynamic motion distractors. In their study, participants searched for a specific shape among an array of six heterogenous shapes that always contained one nontarget shape that was a singleton on some dimension. For different groups of subjects, the distractor was either a static singleton, consisting of a color, size, or fill (i.e., filled vs. outline shape) singleton, or a dynamic singleton, in which the distractor was the only element in motion among other static shapes. It was found that static distractors (i.e., size, color, or fill singletons) could be suppressed in the sense that they were less likely to be fixated by the initial saccade than nonsalient nontarget shapes. Dynamic motion singleton distractors were also avoided, but to a lesser extent—capture was prevented but they were equally likely to be initially fixated as nonsalient nontarget items—suggesting the application of a certain level of inhibition that was able to eliminate their otherwise distracting influence. The ability to suppress a variety of different types of salient stimuli suggests that suppression may be a key component of attentional selection in general.

The present experiments were undertaken prior to the publication of Adams and Gaspelin’s (2024) work. We also were interested in examining avoidance of capture by salient features other than color singletons, and, in particular, we focused on the sudden initiation of motion—motion onset. Motion onset, rather than continuous motion, has specifically been shown to be capable of prioritizing attention in a search task (Abrams & Christ, 2003; Smith & Abrams, 2018). Motion onset can be distinguished from the types of motion studied by Adams and Gaspelin (2024): In their study the motion singleton distractor was in motion from the moment when it first appeared along with the rest of the search array. That is, that element was a new abruptly presented object that happened to be moving. In contrast, motion *onset* comprises the beginning of motion of a preexisting static object. It is thought that motion onset is highly salient because it serves as a cue to animacy (Pratt et al., 2010). Furthermore, some studies have shown that motion (not onset) singletons, similar to those in Adams and Gaspelin (2024), do not capture attention when they are not predictive of the target location (Yantis & Egeth, 1999), which is consistent with the idea that the onset of movement might be critical for moving stimuli to stand out. Also, Abrams and Christ (2003) found faster target identification latencies for target elements that had exhibited a motion onset compared with elements that had been continuously moving from the time that they had first appeared. Thus, motion onset may be even more salient than, or salient in a different way from, motion per se, and is worth investigating in its own right. Also, as far as we know, no one has studied the effects of motion onset in a paradigm in which the motion-onset element is known to never be the target: In the studies that have been conducted, the motion-onset element was not more likely to be the search target than any other item in the display—but it was indeed the target on a subset of trials (e.g., Abrams & Christ, 2003). As a result, it is not even known if motion onset will be distracting when it is known to be irrelevant to the search.

The present experiments differ from those of Adams and Gaspelin (2024) in several important ways in addition to the manipulation of motion onset. First, as in many studies of suppression we included both distractor-present trials as well as distractor-absent trials. Adams and Gaspelin included only the former. Our inclusion of both types of trials permits a quantitative assessment of the magnitude of the distractor effects. Also, the inclusion of distractor absent trials renders the presence of a motion-onset distractor to be unpredictable—perhaps also enhancing its salience somewhat (Becker & Horstmann, 2011; Horstmann & Herwig, 2016; Sayim et al., 2010). Second, Adams and Gaspelin (2024) monitored the initial saccade direction—a measure of overt attentional capture; the present study used complementary behavioral measures that are known to be sensitive to both overt and covert attentional shifts: In addition to search reaction time and accuracy, we also included “probe trials” in one experiment. On probe trials, symbols are briefly superimposed onto some or all of the shapes in the search array, and participants are required to identify as many symbols as possible. The relative rate at which participants report symbols from salient distractors compared to nonsalient distractors serves as an index of the magnitude of suppression. The probe report technique we used is known to provide a fine-grained assessment of attentional processing of individual array elements, and has been used to show suppression of static distractors (e.g., Gaspelin et al., 2015; Kerzel & Renaud, 2023; Lien et al., 2022; Ma & Abrams, 2023b). Third, Adams and Gaspelin (2024) did not separately assess the salience of their different distractors. In the present study, we included an initial experiment to confirm that our motion-onset distractor was indeed highly salient.

In three experiments, we examined whether distractor ignoring based on top-down control can be accomplished for dynamic distractors as it has been shown to occur for static feature singletons. We specifically examined the onset of motion rather than motion per se, because motion onset has been shown to be capable of capturing attention (leading to an advantage during search when the motion-onset element is the target; e.g., Abrams & Christ, 2003). If suppression is a general attentional mechanism that allows inhibition of distracting but salient objects in a scene, then it is possible that people will be able to inhibit capture by motion onset. On the other hand, motion onset might be distinct from other types of salience in attentional priority, perhaps because it is such a strong cue of animacy and may be associated with the evolutionary significance of detecting potential predators in the environment (Pratt et al., 2010). Additionally, it has been shown that, in some cases, suppression of color singletons requires knowledge of the specific color of the distractor (Gaspelin & Luck, 2018b; Oxner et al., 2023; Vatterott & Vercera, 2012; for a review, see Gaspelin et al., 2025). It is not known whether suppression of dynamic distractors may also require knowledge of the specific feature values associated with the distractor, nor is it clear what the relevant feature might be for suppression of an onset of motion. Candidate features include a positive acceleration; detection of “animacy” (e.g., Pratt et al., 2010); or a specific change in an object’s dynamic status (as considered by Chua, 2015). Given that, it seems possible that the sudden initiation of movement may not be easily suppressible.

## Experiment 1

The first experiment served to confirm that our motion-onset distractor was salient and involuntarily captures attention when the search does not permit suppression. This experiment was a variation of the commonly used additional singleton task (Theeuwes, 1992) in which participants searched for a unique shape in a search array, here sometimes in the presence of a motion-onset distractor. Under the attentional set of searching for a unique shape whose identity varies randomly from trial to trial, suppression of salient singleton distractors has been shown to be impossible—in particular, previous studies have shown that color singletons are distracting under these conditions (e.g., Theeuwes, 1992, 2004). Our goal here was to first determine if motion onsets also disrupt search, similar to color singletons, under similar conditions. As noted earlier, in contrast to previous experiments in which the motion-onset element was occasionally the target (and hence might have been intentionally selected), in the present experiment motion-onset elements were always task-irrelevant distractors.

### Method

#### Participants

Twenty-four undergraduate students from Washington University in St. Louis served as participants (additional demographic information about the participants is unavailable). Based on an average effect size of *d*_z_ = 2.3 from Experiment 1 in Gaspelin et al. (2015) that used a similar additional singleton task paradigm, the sample size is sufficient to detect a singleton presence cost with a power of .99 using G*Power (Faul et al., 2007). All participants had normal or corrected-to-normal vision. Informed consent was obtained from each individual.

#### Procedure

**Fig. 1:**
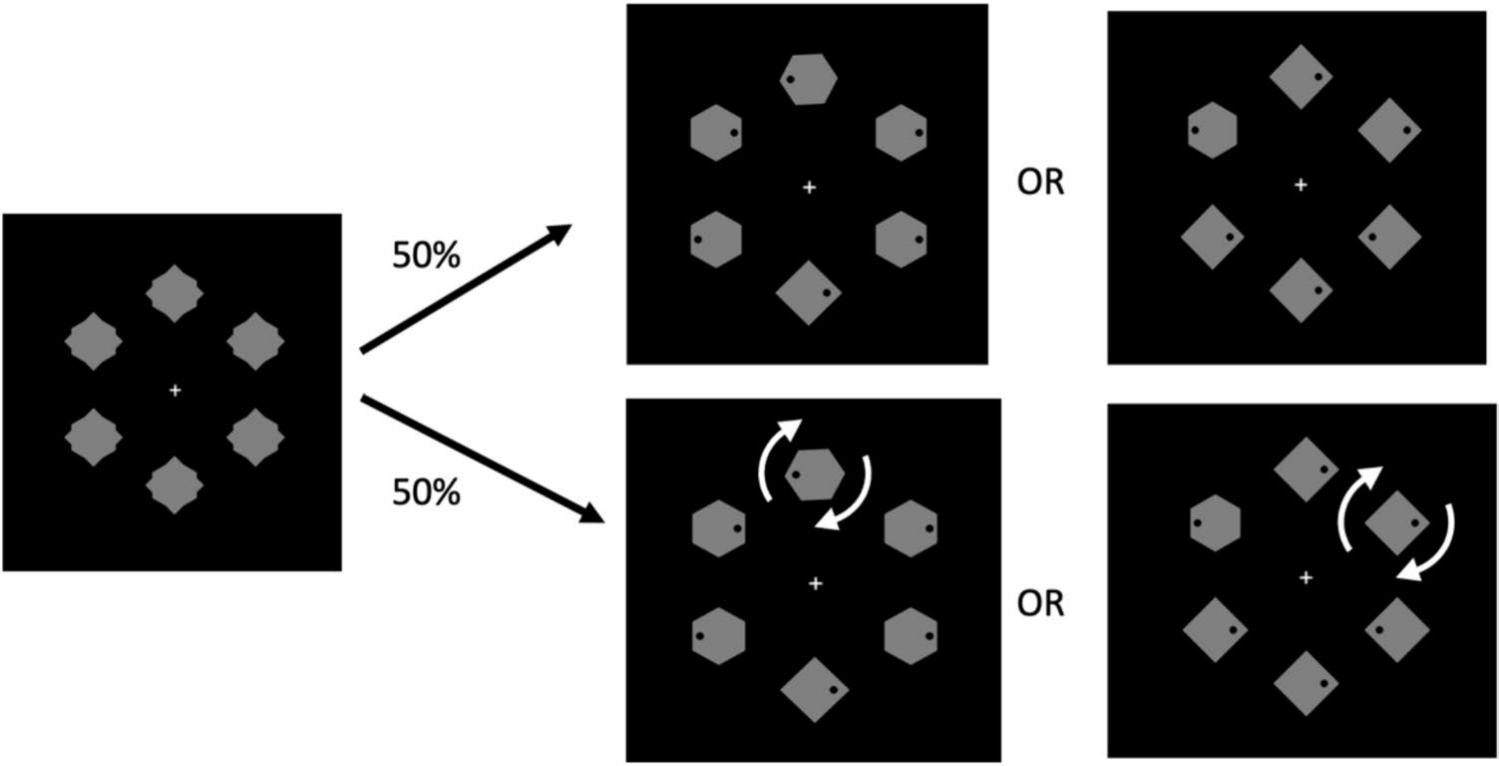
Sequence of events on a trial in Experiment 1, with examples of search arrays. An initial placeholder array contained superimposed diamonds and hexagons. When the search array was revealed, participants indicated the location of the dot inside of the unique shape (either a diamond or a hexagon). On 50% of trials, one nonunique element began to rotate at the time when the search array was revealed (as indicated by arrows in the figure)—a motion-onset distractor

The experiment was programmed and run using PsychoPy software (Peirce et al., 2022). The sequence of events on a trial is shown in Fig. 1. The stimuli were presented against a black background. First, a display of a white fixation cross (height = 0.7°) at the center of an array of six placeholders appeared. The placeholders were equally spaced on an imaginary circle with a radius of 3°. Each shape consisted of a superimposed diamond (1.3° × 1.3°) and hexagon (sized to fit within a 1.6° diameter circle). Two seconds later, the search array was revealed by the removal of one of the superimposed shapes from each placeholder. The search array contained either one diamond and five hexagons or one hexagon and five diamonds, all homogeneously colored in gray. Each shape contained a black dot (0.2° diameter) 0.5° to the left or right of its center. Participants were instructed to search for the unique shape and indicate the location of the dot in it by pressing either the left or right arrow key on the computer keyboard as quickly and accurately as possible.

On one-half of the trials (*motion-onset distractor-present* condition), one of the nontarget shapes began to rotate (clockwise; at a speed of one revolution per second) as soon as the search array was revealed—creating a *motion-onset distractor—*and continued moving for the remainder of the trial.^2^ The dot inside the moving shape remained stationary. Participants were informed that the motion-onset distractor would never be the target shape and thus could and should be ignored for best performance. On the other one-half of the trials (*motion-onset distractor absent*), all shapes in the search array remained stationary. The display of the search array terminated at response, or after 2 s had elapsed without responding. After that, incorrect and missing responses were followed by an “Incorrect!” or “Too slow!” error message for 1 s, respectively. After a blank screen of 1 s, the next trial began.

#### Design

Participants first completed a practice block of 16 trials. The formal experiment contained two blocks of 96 trials each, with a brief break in between blocks. Within each block, each combination of target shape (hexagon or diamond), dot location (left or right), and motion-onset distractor presence (present or absent) occurred equally often, in a random order. The location of the unique shape in the search array and the locations of the dots in the five nontarget shapes were selected randomly.

### Results and discussion

Data analysis was performed using R (R Core Team, 2020). All participants completed the experiment with above 80% accuracy, so all 24 participants were included in the analysis. Trials with reaction times greater than two standard deviations from each participant’s mean reaction time in each condition were excluded from analysis. Trials with incorrect responses were also excluded from reaction time analysis.

**Fig. 2:**
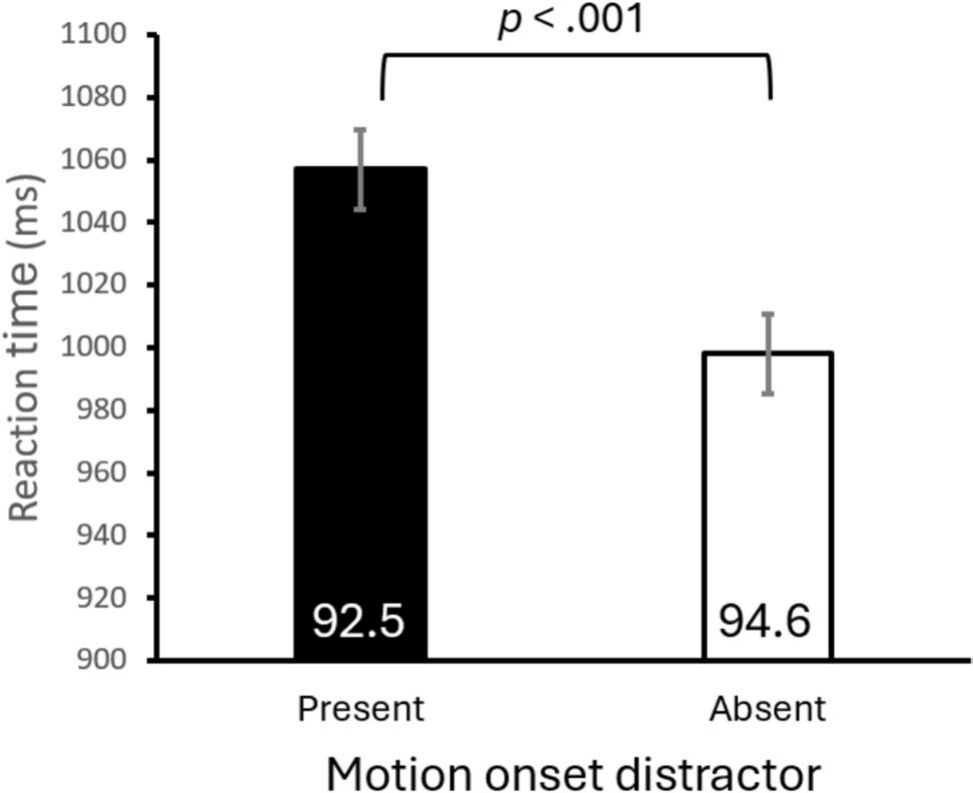
Reaction time and accuracy (percentage correct; the values in the bars) from Experiment 1. The p-value refers to the significant difference in RT. Error bars represent within-subject standard errors (Cousineau et al., 2021)

Reaction times and accuracies are shown in Fig. 2. A paired-samples *t* test revealed that the mean reaction time when a motion-onset distractor was present (1,057 ms) was significantly longer than when the distractor was absent (998 ms), *t*(23) = 4.57,* p* < .001, *d* = 0.41*.* The mean accuracy for distractor-present trials (92.5%) was also significantly lower than that for distractor-absent trials (94.6%), *t*(23) = 2.58*, p* = .017, *d* = 0.38.

The significant increase in reaction time and decrease in accuracy when the motion-onset distractor was present shows that the motion onset did in fact capture participants’ attention as expected, even though the participants were told to ignore the moving shape as it would never be the target. Importantly, the incentive to ignore the motion-onset element contrasts with most prior studies on the effects of motion onset (e.g., Abrams & Christ, 2003; Smith & Abrams, 2018) in which the motion-onset element was occasionally the target of the search. Despite that, participants here were still unable to ignore it. This experiment validates the salience of our manipulation of motion onset. Our next goal was to determine if, under the appropriate conditions, capture by motion onset could be suppressed.

## Experiment 2

Having confirmed that a motion onset could not be ignored when participants searched for a unique shape (as is also the case for color singleton distractors; e.g., Theeuwes, 1992, 1994), we sought here to determine whether capture by motion onset could be suppressed if the search target was a prespecified shape amongst heterogenous shapes. That task does not require attention to any unique elements, and thus permits suppression of singleton distractors: During such searches, color singleton distractors have been found to be suppressed (and their presence even benefits the search performance; e.g., Gaspelin et al., 2015). In their recent study, Adams and Gaspelin (2024) also showed that attentional capture by motion singletons could be prevented: initial saccades did not favor a motion singleton over a nonsalient element.

### Method

#### Participants

A new group of 24 undergraduate students from Washington University in St. Louis served as participants. Based on an average effect size *d*_z_ = .78 from Experiments 2–4 in Gaspelin et al. (2015) that used a similar specific shape search task, the sample size is sufficient to detect a singleton presence effect with a power of 0.96 using G*Power (Faul et al., 2007). All participants had normal or corrected-to-normal vision. Informed consent was obtained from each individual.

#### Procedure

**Fig. 3:**
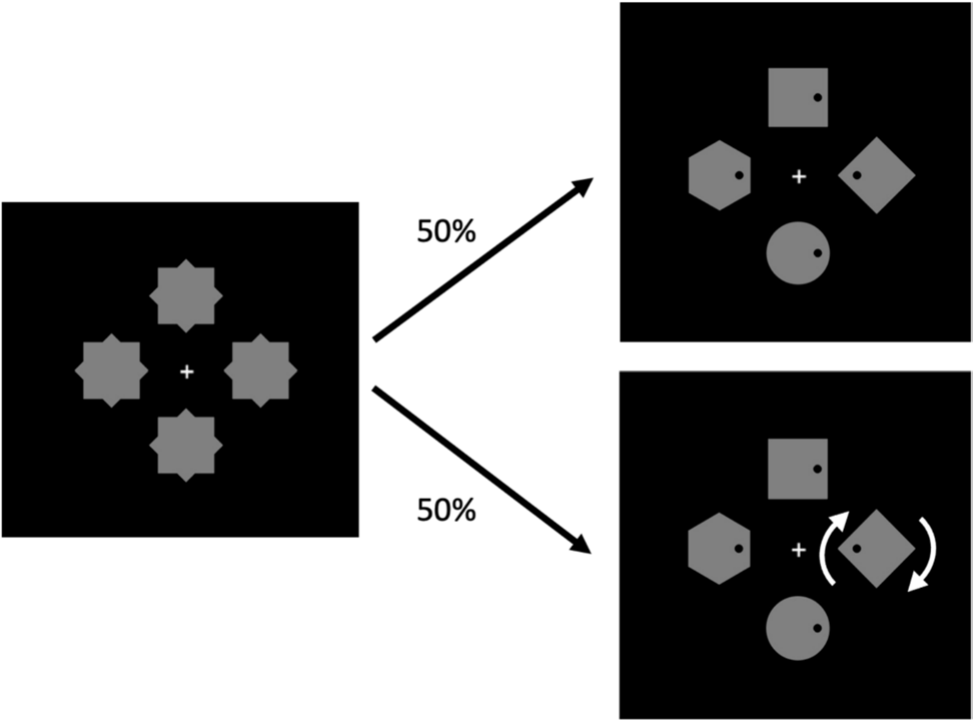
Sequence of events and example search arrays from Experiment 2. An initial placeholder array contained superimposed diamonds and squares. When the search array was revealed, participants indicated the location of the dot inside of the circle. On 50% of trials, one noncircle element began to rotate at the time when the search array was revealed (as indicated by arrows in the figure)—a motion-onset distractor. The search trials in Experiment 3 were identical

The experiment was programmed and run using PsychoPy software (Peirce et al., 2022). The sequence of events is shown in Fig. 3. Each trial began with a white fixation cross that appeared for 1 s followed by four gray placeholders each 2° from fixation in the cardinal directions. We used four-element displays in this experiment because previous research has shown that the RT differences due to the presence of a singleton distractor tends to be greater amongst a smaller set size (Gaspelin et al., 2015, Experiment 2 vs. Experiment 3). Each placeholder consisted of a superimposed square (1.5° × 1.5°) and diamond (1.4° × 1.4°). One second later, the placeholders disappeared, revealing the search array, which consisted of a square (1.5° × 1.5°), a diamond (1.4° × 1.4°), a circle (1.6° diameter), and a hexagon (sized to fit inside a 1.8° diameter circle; the same shapes used by Gaspelin et al., 2015), all colored in gray. Each shape contained a black dot (0.2° diameter) presented 0.5° to the left or right of its center. Participants were instructed to indicate the location of the dot in the circle using the left and right arrow keys on the computer keyboard as quickly and accurately as possible.

On half of the trials, one of the noncircle shapes began to rotate (clockwise; at a speed of one revolution per second) as soon as the search array was revealed—creating a motion-onset distractor. Participants were informed that they could safely ignore the shape in motion, because their target shape, the circle, would never be moving. On the other half of the trials, all shapes in the search array remained stationary. The display of the search array terminated at response, or after 2 s had elapsed without responding. After that, incorrect and missing responses were, respectively, followed by an “Incorrect!” or “Too slow!” error message for 1 s. The next trial began after a blank screen of 1 s.

#### Design

Participants completed a block of 16 practice trials followed by two test blocks of 96 trials each. Fifty percent of the trials contained a motion-onset distractor. The target dot was equally likely to be on the left or the right. The locations of each shape in the search array, the locations of the dots in the nontarget shapes, and the order of trials were random.

## Results and discussion

All 24 participants completed the experiment with more than 80% accuracy and were all included in the analysis. Trials with reaction times more than two standard deviations from each individual participant’s mean reaction time in each condition were excluded from analysis. Trials with incorrect responses were also excluded from reaction-time analysis.

**Fig. 4:**
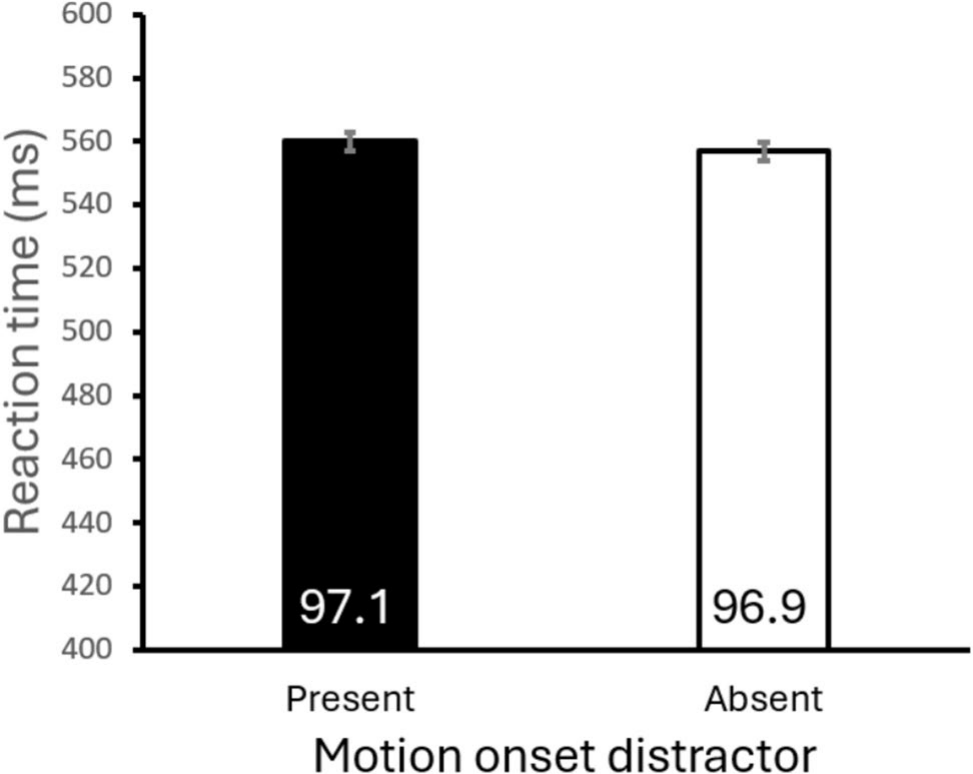
Reaction time and accuracy (percent correct; the values in the bars) from Experiment 2. Error bars represent within-subject standard errors (Cousineau et al., 2021)

Reaction times and accuracies are shown in Fig. 4. A paired-samples *t* test showed that the mean reaction time on distractor-present trials with a motion-onset distractor (560 ms) was not significantly different from the mean reaction time on distractor-absent trials (557 ms), *t*(23)* =* .79*, p =* .436, *d* = .026. The mean accuracy of distractor present trials (97.1%) also did not significantly differ from the accuracy of distractor absent trials (96.9%), *t*(23) = .39*, p* = .698, *d* = .04.

The comparison of mean reaction times for distractor-absent and distractor-present trials shows that the motion-onset distractor did not hinder participant’s performance, revealing avoidance of capture by the motion-onset shape. To confirm the absence of a capture effect, a Bayesian analysis was performed on reaction time and accuracy (using JASP Version 0.17.1; JASP Team, 2020). The analysis confirmed our conclusions, providing moderate evidence for an absence of a difference in reaction times between distractor-present and distractor-absent trials (BF_01_ = 3.51), as well as no difference in accuracy between those two conditions (BF_01_ = 4.35), according to convention (van Doorn et al., 2021).

We also conducted a between-experiment analysis to compare the present results with those from Experiment 1. The analysis revealed main effects of experiment, *F*(1,46) =194.9, *p* < .001, η_p_^2^ = .81, and distractor presence, *F*(1,46) = 21.5, *p* < .001, η_p_^2^ = .32, and most importantly, an interaction between experiment and distractor presence showing that the effects of the motion-onset distractor in the two experiments were indeed different, *F*(1,46) = 18.32, *p* < .001, η_p_^2^ = .28.

The present experiment shows that a motion-onset distractor (which was shown in Experiment 1 to be salient because the presence of a motion-onset distractor impaired the search there) no longer captured attention when the task permitted distractor suppression or avoidance. In particular, the search here was for a specific shape amongst heterogenous shapes. Although there was no benefit when a motion-onset distractor was present—there was also no cost to the search—indicating that the motion-onset distractor had indeed been avoided to some extent in order to offset its otherwise disruptive effect. This finding is consistent with the elimination of overt attentional capture by moving distractors in Adams and Gaspelin (2024). Our results help to reveal a more general inhibitory mechanism that is not limited to salience defined by color singletons or to elements that have been in motion since their initial appearance. However, in both Adams and Gaspelin (2024) and the present experiment, while the dynamic distractors did not capture attention, distractor presence did not yield a benefit to the search as suppressed color singletons have been shown to do. This suggests that different types of salience may invoke different magnitudes of attentional prioritization or require different distractor downweighting mechanisms, which will be further discussed in the General Discussion.

## Experiment 3

Experiment 2 showed that participants were able to inhibit a motion-onset distractor and avoid any cost associated with its presence. Reaction time measures like those used in Experiment 2 offer an overall assessment of performance, but they cannot provide information specifically about attentional shifts to the distractor. This is because reaction time differences could reflect influences other than attentional capture (e.g., filtering costs; Becker, 2007; Folk & Remington, 1998). Our goal in the present experiment was to learn more about the locus of attention during searches that contain motion onsets. To do that we employed a variation of a probe technique similar to that used by Kim and Cave (1995) and others. In that study, the researchers made inferences about the locus of attention based on reaction times for participants to detect probes that were presented briefly on specific elements in the search display. More recently, other researchers have briefly presented letters superimposed onto search elements—with the locus of attention inferred based on the likelihood of participants correctly identifying the letters (e.g., Gaspelin et al., 2015; Kerzel & Renaud, 2023; Lien et al., 2022; Ma & Abrams, 2023b). We take the same approach here. In the present experiment, we repeated Experiment 2 but also included occasional *probe trials* in addition to search trials. On the probe trials, letters were superimposed onto the shapes in the array and participants were required to abandon the search and instead report as many letters as possible. Despite some limitations of the probe-task method (Kerzel & Renaud, 2023; Ma & Abrams, 2025) it can still provide useful information about many aspects of attentional selection.

### Method

#### Participants

A new group of 24 undergraduate students from Washington University in St. Louis served as participants. Based on an average effect size *d*_z_ = 0.94 from Experiment 4 in Gaspelin et al. (2015) that used a similar letter probe method, the sample size is sufficient to detect differences in letter probe report between the motion-onset distractor and the other nontarget shapes with a power of 0.99 using G*Power (Faul et al., 2007). All participants had normal or corrected-to-normal vision. Informed consent was obtained from each individual.

#### Procedure

**Fig. 5:**
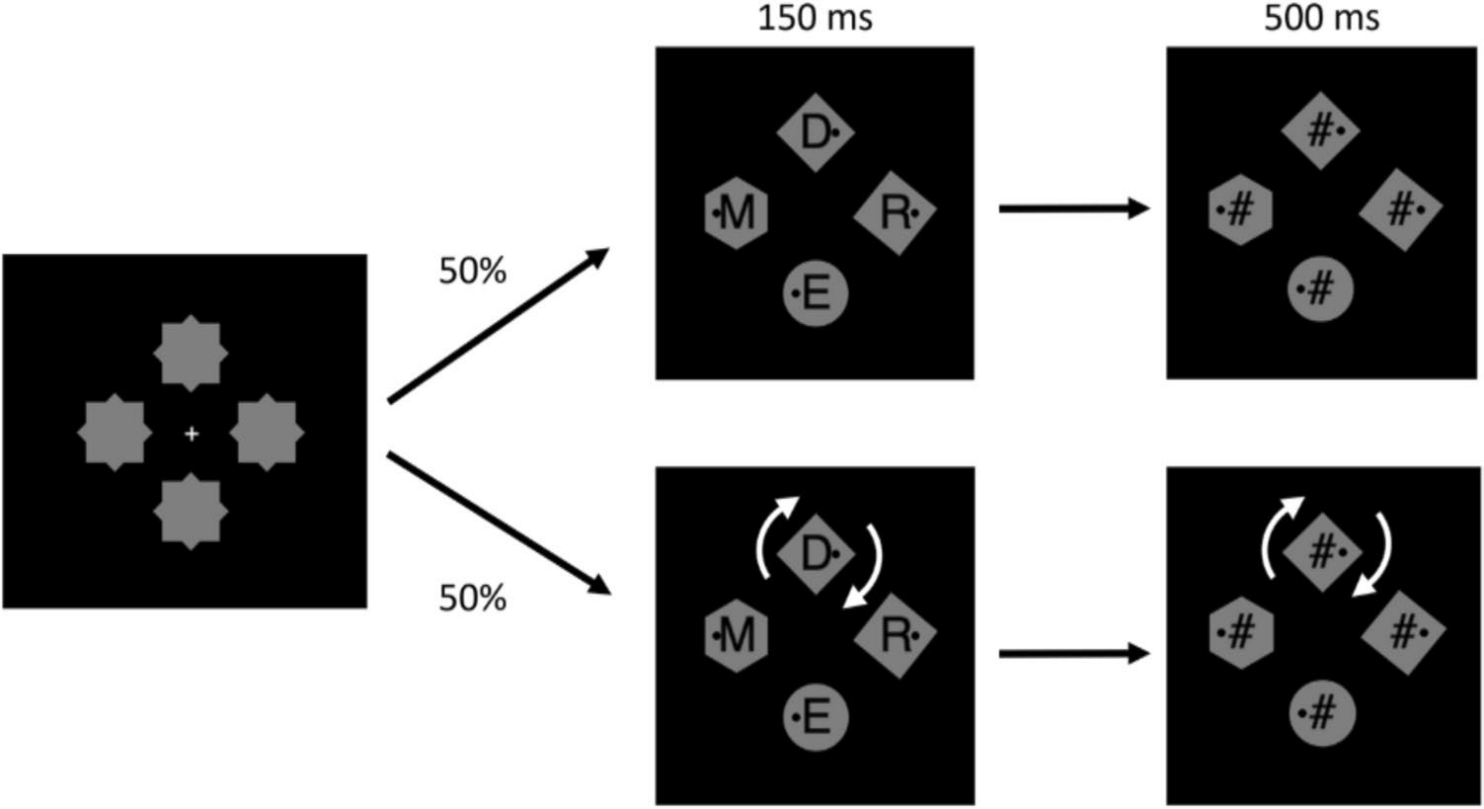
Sequence of events on the probe trials (1/3 of total trials) from Experiment 3. When the search array was revealed, each shape contained a letter for 150 ms, followed by a mask. Participants abandoned the search on such trials and instead identified as many letters as possible. On 50% of the trials, one noncircle element began to rotate at the time when the search array was revealed (as indicated by arrows in the figure)—a motion-onset distractor. The search trials (2/3 of total trials, interleaved randomly with probe trials) were identical to those from Experiment 2, shown in Fig. 3

The experiment contained search trials and probe trials. The search trials were identical to those from Experiment 2. The sequence of events on probe trials (which comprised one-third of the total trials) is shown in Fig. 5. On the probe trials, when the search array was revealed, each shape contained a black letter (1.2° in height) superimposed on it. Participants were instructed to abandon the search on such trials and to identify as many letters as possible. The letters were visible for 150 ms and were then each replaced by a pound sign mask (1.2° in height) for another 500 ms. The search array was followed by a response selection screen displaying an alphabet. Participants used a computer mouse to select as many letters as they recalled, in an unspeeded manner. As on the search trials, on 50% of the probe trials, one noncircle element began to rotate (clockwise; at a speed of one revolution per second) at the time when the search array was revealed (as indicated by arrows in the figure)—a motion-onset distractor. The letter inside the rotating shape remained stationary.

#### Design

Participants first completed a practice block of 24 search trials. Next, participants were instructed about the probe task, and completed another practice block of 30 trials that contained interspersed probe trials and search trials. The formal experiment consisted of two blocks of 144 trials each. Each block contained 96 search trials and 48 probe trials. A motion-onset distractor was present on 50% of the search trials and 50% of the probe trials. The four letters on each probe trial were randomly selected without replacement from the 26 letters in the alphabet.

### Results

Reaction times that were more than two standard deviations away from each individual’s mean reaction time in each condition were removed from the analysis. Trials with incorrect responses were also excluded from reaction time analysis. All participants had greater than 80% search accuracy and reported at least 0.8 letters per probe trial, and were included in the analysis.

#### Search-task results

**Fig. 6:**
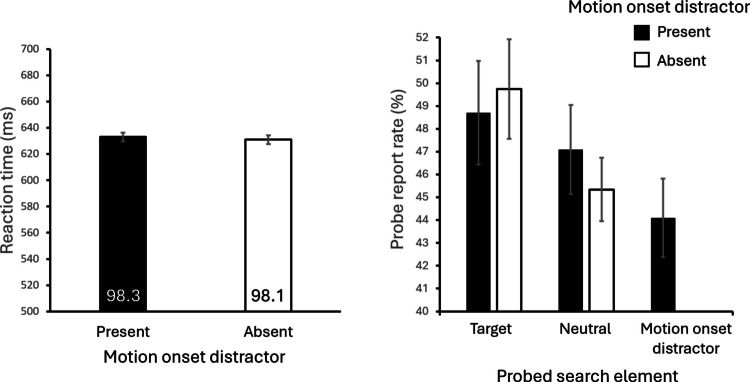
Left panel: Reaction time and accuracy (percent correct; the values in the bars) from the search trials of Experiment 3. Right panel: Probe report rates for each possible probe letter location. (*Neutral* denotes the static nontarget shapes.) Error bars represent within-subject standard errors (Cousineau et al., 2021)

Reaction times and accuracies are shown in Fig. 6 (left panel). A paired-samples *t* test showed that the mean reaction time for motion-onset distractor present trials (633 ms) was not significantly different from the mean reaction time for distractor absent trials (631 ms), *t*(23) *=* .42*, p =* .676, *d* = 0.09. The mean accuracy on distractor-present trials (98.3%) was also not different from the accuracy on distractor-absent trials (98.1%), *t*(23) = .35*, p* = .732, *d* = 0.07. These results replicate the findings from Experiment 2 showing that the salient motion-onset distractor did not impair performance and thus appears to have been avoided.

To confirm the absence of a capture effect, again a Bayesian analysis was performed on reaction time and accuracy (JASP Team, 2020). The analysis confirmed the null results, providing moderate evidence for an absence of a difference in reaction times between distractor-present and distractor-absent trials (BF_01_ = 4.29), as well as no difference in accuracy between those two conditions (BF_01_ = 4.41; van Doorn et al., 2021).

#### Probe-task results

Probe letter report rates are shown in Fig. 6 (right panel). Participants reported an average of 1.99 letters per trial. Of the letters reported, 93.5% of them were present in the search array. The average number of letters reported on distractor absent trials (1.99 letters) was not significantly different from the average number of letters reported on distractor present trials (2.00 letters), *t*(23) = .65, *p* = .523, *d* = 0.13.

The probe letter report rate, referring to the mean probability of correctly reporting the presented letter on a given item, was computed for each type of stimulus. For trials where the motion-onset distractor was present, letter report rate of the distractor (44.1%) was no higher (actually numerically lower) than the static nontarget shapes (which we refer to as *neutral* shapes, 47.1%), *t*(23) = 1.48, *p* = .151, *d* = 0.3. This suggests an equivalent amount of attention allocated to the motion-onset distractor and a nonsalient distractor, consistent with the idea that attentional capture by motion onset was eliminated. A Bayesian analysis (JASP Team, 2020) is consistent with the null difference in letter report rates for the distractors and neutral stimuli for distractor present trials (BF_01_ = 1.78; van Doorn et al., 2021).

A 2 × 2 analysis of variance (ANOVA) was performed to analyze the effects of motion-onset presence (present vs. absent) and stimulus identity (target vs. neutral shape) on probe letter report rates, excluding data from the motion-onset distractor location. There was a marginally significant main effect of stimulus identity, *F*(1,23) = 3.33, *p* = .081, η_p_^2^ = .13, with the mean probe letter report rate on target shapes (49.2%) marginally higher than for the neutral shapes (46.2%). This is consistent with the task requirement to search for the target shape. There was no main effect of motion-onset presence, *F*(1,23) = .10, *p* = .75, η_p_^2^ = .00, nor was there an interaction between the two factors, *F*(1,23) = .94, *p* = .342, η_p_^2^ = .04, consistent with avoidance of capture by the motion-onset distractor.

### Discussion

The present results confirm and extend the findings of Experiment 2. The motion-onset distractor had no disruptive effect on the latency or accuracy in the search task, consistent with effective avoidance of the salient distractor. Converging evidence comes from the probe task, in which we assessed attentional allocation to individual array elements. There, we confirmed that the highly salient motion-onset distractor did not receive greater attentional priority than a nonsalient neutral shape. Each of these results indicates that the salient motion-onset singleton was successfully ignored.

## General discussion

In the present study, we examined attentional avoidance or suppression of salient motion onsets, to supplement and extend most previous studies of suppression that have examined color singletons. Experiment 1 revealed, using an additional singleton task that was not expected to permit suppression, that motion onset is indeed salient—and cannot be ignored even when known to be irrelevant to the search. Experiments 2 and 3 employed search tasks in which the target was a prespecified shape—a situation known to permit suppression of color singletons—and revealed that salient motion onsets could indeed be ignored because their presence did not interfere with the search. The findings suggest the existence of a general inhibitory mechanism involved in attentional selection that is not limited to one particular type of salient stimulus (viz. color singletons).

### Distractor suppression versus avoidance

While we have found that participants were able to avoid any adverse effects of motion onsets (when searching for a specific shape amongst heterogenous shapes), the impact of motion-onset distractors was not reduced below a neutral baseline—that is, there was not a distractor presence advantage—often considered to be indicative of “attentional suppression” (Liesefeld, et al., 2024). If it occurs, below-baseline attentional suppression would imply the operation of an active mechanism that effectively downweights the attentional priority of salient distractors. Instead, it might be more appropriate to characterize the present results as revealing an ability to “avoid,” “ignore,” or “eliminate capture” by motion onsets. It is important to note, however, that the present findings do not rule out the involvement of an active suppressive mechanism. That is because our reaction time and probe report results reflect the combined effects of any attentional capture plus any suppression or inhibition. As a result, it is not possible for us to distinguish between (a) a substantial capture effect and an equivalent strength of suppression compared with (b) no capture and no active suppression. Both scenarios would lead to the absence of an effect of a motion-onset distractor, as we observed. Distinguishing between those possibilities will require other research methods—with neuroscientific techniques being likely to be helpful (e.g., Gaspelin & Luck, 2018a; Ma et al., 2025; Sawaki & Luck, 2010).

### Static versus dynamic distractors

A recent study by Adams and Gaspelin (2024) also examined avoidance or suppression of salient features other than color singletons. They, like us, found that certain types of salient dynamic stimuli could be rendered innocuous—with their presence having no impact on the search (as indexed by the initial saccade direction in Adams and Gaspelin, and by search reaction times and probe letter report rates in the present study). Thus, our findings using reaction time methods conceptually replicated and supplemented their evidence from eye tracking. Our results, as well as those of Adams and Gaspelin (2024), contrast somewhat with earlier findings involving static salient stimuli, such as color singletons. Static salient stimuli have been shown to be able to be suppressed below “baseline” in the sense that they were less likely to be fixated than nonsalient nontarget elements (Adams & Gaspelin, 2024), or their presence actually facilitated identification of the search target (Gaspelin et al., 2015; Ma & Abrams, 2023c).

One possible explanation for the inability to suppress dynamic distractors below baseline is that the dynamic stimuli studied may simply have been more salient than static ones. If the suppressive mechanism has limited strength, it may not be powerful enough to offset highly salient stimuli. However, one aspect of Adams and Gaspelin’s (2024) findings argues against that interpretation: They found that participants were capable of suppressing below baseline a compound stimulus that was both a static (color) and dynamic (motion) singleton. Such a stimulus seems likely to have been at least as salient as a motion singleton alone, so what might be critical to permit suppression is the ability to select the to-be-avoided element on the basis of static information (as suggested by Adams & Gaspelin, 2024).

Another explanation for the inability to suppress dynamic stimuli below baseline stems from several key differences between static attributes, such as color, and dynamic attributes. First, for suppression to prevent attentional capture from occurring, the feature signaling the distractor must be able to be accessed and processed rapidly. Color can be easily processed during preattentive viewing (as in the “rapidly extracted attributes” of Wolfe, 2021), and as many researchers have shown does indeed permit suppression (e.g., Gaspelin et al., 2015; Sawaki & Luck, 2010). In contrast, dynamic elements in a scene, by definition, require a certain amount of time to be distinguishable from static elements. This additional time requirement may preclude the proactive suppression of dynamic stimuli. Second, using search tasks similar to those in the present study, a number of studies have shown that suppression relies on a feature-based inhibitory mechanism that can only downweight distractors consistently appearing in a known color. Distractors that randomly change colors, or even only alternate between two colors cannot be suppressed (Gaspelin & Luck, 2018b; Oxner et al., 2023; Vatterott & Vercera, 2012). Several cases of suppression of color-unpredictable distractors have been reported—but those employed tasks that required subitizing instead of visual search (Drisdelle et al., 2025; Ma & Abrams, 2023a, 2023b). This implies that, at least in visual search tasks, the distractor template for inhibitory control has a limited single-slot capacity for a specific feature value. It is possible that dynamic motion cannot be perceptually represented as a single consistent feature, as there are multiple feature values (e.g., speed, acceleration, moving direction) simultaneously signaling its salience.

Last but not least, the mere attenuation, but not below-baseline suppression, of dynamic distractors might also be explained by habituation, rather than active attentional inhibition. Habituation refers to a general learning principle in which an organism’s response to a stimulus decreases with repeated exposure (see a review by Turatto, 2023). In the context of attentional capture, as the observer learns the regularities in repeated stimuli and generates predictions for upcoming ones, orienting is found to be attenuated to stimuli matching predictions (Bonetti & Turatto, 2019; De Tommaso & Turatto, 2019; Folk & Remington, 2015; Turatto et al., 2018). Consequently, habituation might reduce attentional capture by irrelevant distractors, but, due to the absence of active inhibition, habituation would not be expected to lead to a benefit of singleton distractor presence. Habituation has been proposed as a possible explanation for the effects of static salient distractors that fail to be suppressed below baseline but do lead to reduced capture over time, such as unpredictable color singletons (Sawaki & Luck, 2010; Vatterott et al., 2018). In those studies and the present one, the extent to which habituation contributed to the attenuation is unknown, and would be an important question to examine in future work.

### Limited effects of dynamic distractors

The present results contribute to a growing number of findings that have shown limited behavioral effects of dynamic distractors despite their apparent salience. For example, Hillstrom and Yantis (1994) found that moving elements in a search could be rapidly and efficiently selected when they were predictive of the target location—showing that they were highly salient. But the same stimuli did not capture attention when they were uncorrelated with the target location—showing that they could be avoided. Similar results were reported by Yantis and Egeth (1999). And Chua (2015) found that capture by dynamic stimuli depended to a great extent on the observer’s attentional control settings—revealing the ability to avoid them, under the right circumstances.

### Motion versus motion onset

Previous studies have revealed a distinction between moving elements (objects that are in motion from the moment that they appear) and those that are initially static and then begin to move. Targets that are in (Abrams & Christ, 2003) or near (Smith & Abrams, 2018) motion-onset elements are prioritized relative to ones in or near elements in continuous motion. Motion onset, but not continuous motion, has also been shown to disrupt inhibition of return (Abrams & Christ, 2005). Given these results, we had speculated that motion onsets might have been especially challenging to avoid—however, the present findings indicate instead that avoidance of capture by motion onset is very similar to that of motion in general. In particular, it was possible for participants in the present study to avoid deleterious effects of motion onset, but it was not possible for them to suppress the motion-onset element below baseline. This pattern is similar to what Adams and Gaspelin (2024) found for continuous motion. Taken together the results suggest that attentional suppression may depend not exclusively on the salience of the stimuli in question, but also on the extent to which the relevant stimulus feature can be rapidly processed and represented in a form that permits the inhibitory operation.

### Target-feature upweighting

There is some evidence that attentional selectivity may sometimes involve not only suppression of irrelevant stimuli, but also enhancement or upweighting of relevant ones. For example, Chang and Egeth (2019) showed that people not only are slower to detect stimuli in colors known to be irrelevant to the search, but they are faster to detect stimuli in known target colors, relative to neutral colors. While we have shown that capture by motion onset appears to be avoidable, it is not known to what extent enhancement of the static elements in the display might have played a role. That would be a useful question to address in the future.

## Conclusion

The present experiments show that people have the ability to avoid distraction by highly salient motion-onset stimuli when such stimuli are known to be irrelevant to the search goal. The results not only expand the understanding of attentional avoidance, which appears to be capable of inhibiting capture by a variety of static and dynamic features, but they also reveal its limitations, suggesting that dynamic stimuli may be fundamentally different from static stimuli in terms of prioritizing attentional processing.

## Data Availability

Trial-level data and analysis scripts for all experiments are available online (https://osf.io/uhz76). Other experiment materials and code are available upon request. None of the experiments was preregistered.

## Footnotes

1 Following the terminology proposed by Liesefeld et al. (2024), in this paper when describing results, we use “suppression” (of a salient distractor) to refer to a reduction in measured attentional priority below a well-defined baseline (specifically a condition with a neutral distractor in place of a salient one). The terms “avoidance,” “ignoring,” or “elimination of capture” by a salient distractor refer to cases where the distractor does not significantly capture attention but is also not inhibited below the aforementioned baseline.

2 It is important to point out that the motion onset distractor not only began to move—but it was also a motion singleton. That is, after the motion had started, the motion onset element was the only element in the search array that was moving—all of the other elements were static. Thus, conclusions about motion onsets here may apply to dynamic stimuli in general.

## Code availability

Experiment programs and code are available upon request.

## Declarations

### Open practices statement

Trial-level data and analysis scripts for all experiments are available online (https://osf.io/uhz76). Other experiment materials and code are available upon request. None of the experiments was preregistered.
